# Investigation of the reproductive behavior of Tarim pigeons

**DOI:** 10.5194/aab-68-395-2025

**Published:** 2025-06-19

**Authors:** Lin Zhu, Mahmoud Kamal, Mengyue Sun, Yao Li, Rui Fu, Mohamed E. Abd El-Hack, Zewu Wang, Kailun Yang, Fengming Li, Yanfen Cheng

**Affiliations:** 1 College of Animal Science, Xinjiang Agricultural University, Ürümqi, 830052, China; 2 Laboratory of Gastrointestinal Microbiology, National Center for International Research on Animal Gut Nutrition, Nanjing Agricultural University, Nanjing, 210095, China; 3 Animal Production Research Institute, Agricultural Research Center, Dokki, Giza 12618, Egypt; 4 Department of Industrial Pharmacy, College of Pharmaceutical Sciences and Drug Manufacturing, Misr University for Science and Technology (MUST), P.O. Box 77, Giza, Egypt; 5 Poultry Department, Faculty of Agriculture, Zagazig University, Zagazig 44511, Egypt; 6 Xinjiang Kunlun Cuiling Pigeon Industry Co. Ltd, Kashgar, 844000, China

## Abstract

This study aims to investigate pigeon behavior across various stages of the breeding period and record their behavioral frequencies to contribute to the theoretical foundation for pigeon breeding and management. The experiment involved 10 pairs of pigeons in the incubation period, 9 pairs of parent pigeons within the first 10 d of the nurturing period, and 9 pairs of parent pigeons with chicks older than 10 d. A direct observation method was used to record behaviors from 08:22 to 22:00 LT daily. Our results revealed that the female pigeon was primarily responsible for incubation during the incubation period. The average incubation duration for female pigeons was 7.94 ± 0.39 h, whereas the average incubation duration for male pigeons was 4.05 ± 0.63 h. The results showed significant differences ( P<0.001 ) in the amount of feed intake (FI) between the three aforementioned groups. The highest FI was found in the group with chicks older than 10 d (A10D), followed by the group within the first 10 d of the nurturing period (W10D), and then the incubation period (IP) group. Additionally, the results showed significant differences ( P<0.05 ) in the frequencies of water and grit consumption and resting behaviors. Drinking water, grit consumption, and resting were highest in the A10D group, followed by the W10D group, and then the IP group. However, the frequencies of ingestion, nurturing, stretching, and preening behaviors did not show significant differences during the studied periods. The findings indicate that there were significant differences ( P<0.05 ) in courtship and fighting behaviors, with the most fighting observed in the W10D period, followed by the A10D period, and then the IP period. The most courtship behavior was observed in the A10D period, followed by the W10D period, and then the IP period. The study did not find any significant differences in mating behavior during these periods. Given that behaviors such as FI, drinking water, eating grit, resting, and courtship were highest during the A10D period, care should be taken during this period, especially regarding the provision of necessary care, in order to enhance economic profit and maintain the health of the pigeons.

## 1 Introduction

The term “pigeon” refers to a member of the enormous Columbidae family, which inhabits temperate and tropical regions globally and is classified under the order Columbiformes (Akter et al., 2020). Pigeons are one of the most important commercial poultry species due to the quality of their meat, which is rich in proteins, minerals, and vitamins. They are also considered ornamental birds (Muraduzzaman et al., 2023). Pigeons mate at 5–7 months of age, with males and females being equally responsible for nest construction. Egg incubation is a crucial process in the early stages of a baby pigeon's life, involving both the male and female parents' involvement (Shariar, 2020).

With respect to egg incubation, Murrell (2020) indicated that breeding males occupy the daytime shift in intervals (09:30–13:30 and 15:30–17:30 LT), whereas females predominantly undertake the late afternoon to mid-morning shift (about 17:30–09:30 LT). Pigeons exhibit distinct mating and brooding behaviors compared to other domestic birds, as their young squabs are entirely reliant on their parents for feeding and care. Both parents exhibit an increase in aggressive behavior during brooding after the eggs have hatched (Mohamed et al., 2016; Kozlowski et al., 2016). Pigeons construct two nests for the purpose of rearing their squabs, using each nest alternately for egg incubation and the care of hatched squabs, thereby maintaining a meticulously planned breeding cycle. Pigeon eggs require around 17–18 d of incubation to hatch. When the squabs reach approximately 15 d of age, the female parent may commence laying new eggs for the subsequent breeding cycle; therefore, the male parent predominantly assumes the responsibility of feeding the existing squabs (Adawy and Abdel-Wareth, 2023; Wang et al., 2023).

Pigeons typically build two nests to rear their squabs, alternating between each nest for incubating eggs and nurturing newly hatched squabs in successive cycles. Pigeons are altricial birds, meaning that the young cannot feed themselves after hatching and must depend on the crop milk provided by the parent pigeons, which is delivered in a mouth-to-mouth manner by both the male and female parents (Mahdy, 2021; Jin et al., 2023). Understanding parental behavior toward their squabs is essential to meet the squabs' nutritional needs. These nourishments must fulfill the squabs' requirements to ensure an enhanced body weight, growth rate, and overall positive health indicators (Dong et al., 2013; El Shoukary and Mousa, 2018). Pigeons' crop milk and reproductive behavior (such as nest defense, egg incubation, and brooding squabs) are associated with the prolactin level (Gillespie et al., 2012). Prolactin aids in the nesting behavior of parents, which in turn enhances their feeding solicitations, specifically the squab-oriented bill opening (Skrade et al., 2017). Given the essential function of crop milk in squab development, nutritionists have focused much attention on it, and investigations into its composition and nutrient profile continue (Jin et al., 2023).

In a single reproductive cycle, a pair of pigeons can rear a maximum of two squabs. Commercial production employs manual incubation procedures to enhance production efficiency, enabling a pair of breeding pigeons to rear more than two squabs, thereby resulting in increased economic benefits (Abdel Fattah et al., 2019; Zhang et al., 2023). Furthermore, Tang et al. (2019) demonstrated that the quantity of reared squabs significantly influences the survival rate of 28 d old squabs, the egg-laying interval, and variations in the parent pigeons' body mass. On the other hand, Gharib et al. (2024) discovered that increasing the care of squabs or eggs incubated did not have an adverse effect on adult behavior or squab performance. The behavior of the parent pigeons during the breeding period not only comprises hatching, eating, feeding, drinking, preening, and resting but also familiar behaviors such as the parents combing each other's feathers (Wang et al., 2023).

The objective of this study was to investigate pigeon behaviors and improve the rearing management of caged pigeons to increase their efficiency and production.

## 2 Material and methods

### 2.1 Experimental animals

The experiment was conducted at Xinjiang Kashgar Kunlun Cuiling Pigeon Industry Co., Ltd. in Shufu County, Kashgar Prefecture, Xinjiang. The experiment involved 10 pairs of pigeons in the incubation phase (IP), 9 pairs of parent pigeons with offspring younger than 10 d (W10D), and 9 pairs of parent pigeons with offspring older than 10 d (A10D). Blinding techniques were used to record and analyze all behavioral data in order to reduce observer bias.

### 2.2 Experimental design

Pigeons were reared in a three-level, 60cm×50cm×50cm metal cage. During the experiment, sunrise was at 08:06–08:16 LT (local time), sunset was at 21:40–21:54L̇T, daytime began at 07:38–07:48 LT, and nighttime began at 22:08–22:23 LT. The experiment was conducted in August. Pigeons have poor vision at night. Based on the observation of pigeons' behavior during the pre-experiment period and on the local sunrise and sunset times, the final recording period was set from 08:22 to 22:00 LT. The recording equipment was a HIKVISION surveillance camera (model: 3T27EWDV3-L8 mm).

The total number of occurrences of various behaviors during different periods of the breeding parent's activity period was documented by taking the beginning of an action to the end of the action as one repetition and by taking 1 h as a time unit to count the frequency. As the activity time of pigeons from 08:22 to 09:00 LT was less than 1 h, the behavioral frequency from 08:22 to 10:00 LT was combined and calculated and then converted into the behavioral frequency per hour.

### 2.3 Behavioral observation methods

The frequency of each behavioral state was recorded across time using direct observations. The behavioral parameters for this experimental study were established by drawing upon the research conducted by Kozlowski et al. (2016) and by conducting pilot studies to construct a comprehensive behavioral spectrum. A direct observation technique was utilized to document the frequency of individual behavioral states during various time intervals. The ethogram is illustrated in Table 1.

**Table 1:** The observed behavioral habits of pigeons.

Behavior	Description
Stretching	The parent pigeon displays flight by extending one leg and wing simultaneously, elevating both wings, waving their wings, or trembling their body.
Resting	There are two ways for the parent pigeon to rest (lying down or standing up). Bending both legs, settling on the abdomen, occasionally putting one wing beneath their body, relaxing their head and neck, and gently closing their eyes are the key characteristics of recumbent behavior.
Standing	Standing and resting involves placing both legs on the ground or, occasionally, placing only one leg on the ground and bending the other leg toward the abdomen.
Preening	The parent pigeon displays grooming behavior by using their beaks to preen their neck, abdomen, tail, legs, wings, and surrounding feathers.
Courtship	The pigeons groom and feed each other, with mutual grooming mainly around the head and eye area.
Fighting	The parent pigeon uses its beak to peck at the head and other parts of the body of other pigeons.
Nurturing	The parents and squabs engage in mouth-to-mouth feeding during daytime observation periods.
Mating	The male pigeon engages in mating by raising its neck, walking around the female pigeon, squatting on her back, spreading his wings, and pressing down his tail feathers.
Eating grit	Pigeons feed on healthy sand, etc.

The experimental birds consisted of Tarim pigeons from the same pigeon house, and the pigeon house and pigeon cage were thoroughly disinfected before the experiment. Each monitoring device was assigned to one pair of pigeons and recorded the frequency of ingestion, water consumption, grit consumption, nurturing, stretching, preening, fighting, courtship, and mating behavior during the incubation and nursing period. The parent pigeons used in the experiment were fed with mixed raw grains (corn, wheat, sorghum, peas, etc.) at 12:00 LT. The pigeons were provided with feed, sand, and water ad libitum. During the experiment, the pigeons had visual access to other breeding pairs. The animals were raised under one environmental condition.

### 2.4 Statistical analysis

After manipulating the experimental data with Microsoft Excel 2021, we analyzed the data using the IBM SPSS Statistics 26.0 (SPSS, 2019) software. To compare the male and female incubation times, we employed a one-sample t test. We used a one-way ANOVA to compare the behavioral frequency across different periods and then utilized the Duncan test for multiple comparisons. The examination findings are reported as the mean ± standard error, with a P value<0.05 signifying a significant difference.

## 3 Results

### 3.1 Time and frequency of rotation of brood pigeons during the incubation period

**Figure 1:**
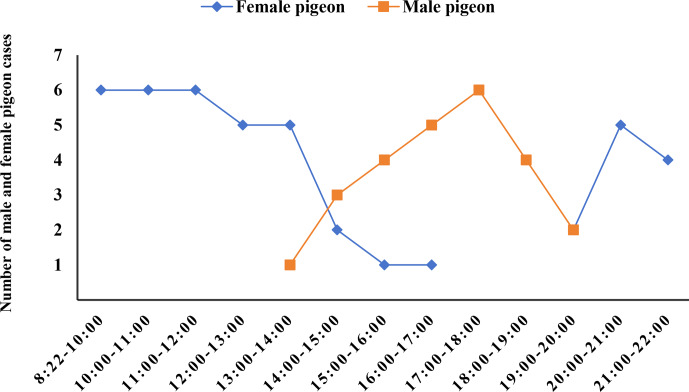
Incubation time for male and female pigeons. This figure illustrates the number of females and males incubating eggs in 1 h intervals.

The results in Table 2 display brood pigeons' rotation time and frequency during the incubation phase. Both male and female pigeons alternated incubating eggs throughout this time. Female pigeons hatched all of their eggs between 08:22 and 13:00 LT, with males dominating the incubation period from 13:00 to 20:00 LT, while females continued to incubate the eggs after 20:00 LT. In contrast, males and females frequently alternated for brief periods between the hours of 12:00 and 13:00, 14:00 and 16:00, and 19:00 and 22:00 LT, as shown in Fig. 1.

**Table 2:** Average values of incubation time and frequency for male and female pigeons.

	Exchange frequency	Incubation time of	Incubation time of	P value
	(counts)	female pigeons (h)	male pigeons (h)	
Mean	7.44 ± 1.17	7.94 ± $0.39^{a}$	4.05 ± $0.63^{b}$	0.001

^a,b^ Means within the same column differ substantially ( P<0.001 ).

The average incubation period was 7.94 ± 0.39 h for females and 4.05 ± 0.63 h for males. Females took 96.05 % longer to incubate than males ( t=5.273 , P<0.05 , N=10 ; Table 2).

### 3.2 Feed intake of parent pigeons at different stages of the breeding period

Table 3 illustrates the feed intake (FI) of parent pigeons during various phases of the breeding period. There was a big difference between the amount of FI in the nursing period and the amount eaten during the incubation period ( 55.09 ± 3.35 , P<0.05 ). The difference was 76.25 % ( P<0.05 ) with respect to the FI of parents with offspring that were less than 10 d of age ( 97.10 ± 5.56 , P<0.05 ) and more than 10 d of age ( 107.14 ± 4.79 , P<0.05 ).

**Table 3:** Comparison of pigeon feed intake during different phases of the breeding period.

Breeding season	IP	W10D	A10D	P value
Feed intake	55.09 ± $3.35^{b}$	97.10 ± $5.56^{a}$	107.14 ± $4.79^{a}$	0.001

^a,b^ Means within the same column differ substantially ( P<0.001 ). Abbreviations used in the table are as follows: IP – incubation period; W10D – within the first 10 d of the nurturing period; A10D – after 10 d during the nursing period. Values in the IP, W10D, and A10D columns are given in grams per day per pair.

### 3.3 The frequency of ingestion, water consumption, grit consumption, and nurturing behavior among pigeons during different stages of the breeding period

Table 4 displays the frequency of ingestion, water consumption, grit consumption, and nursing behavior of pigeons during various periods of the breeding season. According to our findings, the disparities in ingestion across breeding times were not statistically significant. During the nursing period ( 100.22 ± 13.76 , P<0.05 ) and the first 10 d of the nurturing period (W10D) ( 88.00 ± 19.05 , P<0.05 ), there was a higher frequency of grit consumption than during the incubation phase ( 46.80 ± 6.99 , P<0.05 ). Within the first 10 d of the nurturing period ( 37.11 ± 4.32 P<0.05 ) and incubation period ( 20.90 ± 2.46 , P<0.05 ), parental water intake was lower than during A10D: water consumption during the nursing period (A10D) ( 53.78 ± 4.38 P<0.05 ) was much better, with 44.92 % ( P<0.05 ) more frequent water consumption and 157.32 % ( P<0.05 ) more frequent water consumption than the W10D and IP periods, respectively.

**Table 4:** Comparison of the total frequency of ingestion, water consumption, grit consumption, and nurturing behavior among pigeons during different phases of the breeding period.

Total frequency	IP	W10D	A10D	P value
Ingestion	65.30 ± 11.64	103.0 ± 8.25	104.0 ± 19.64	0.093
Drinking water	20.90 ± $2.46^{c}$	37.11 ± $4.32^{b}$	53.78 ± $4.38^{a}$	0.001
Eating grit	46.80 ± $6.99^{b}$	88.0 ± $19.05^{a}$	100.22 ± $13.76^{a}$	0.026
Nurturing	–	13.44 ± 1.57	13.11 ± 1.34	0.87

^a,b,c^ Means within the same column differ substantially ( P<0.05 ). Abbreviations used in the table are as follows: IP – incubation period; W10D – within the first 10 d of the nurturing period; A10D – after 10 d during the nursing period. Values in the IP, W10D, and A10D columns are given in grams per day per pair.

**Figure 2:**
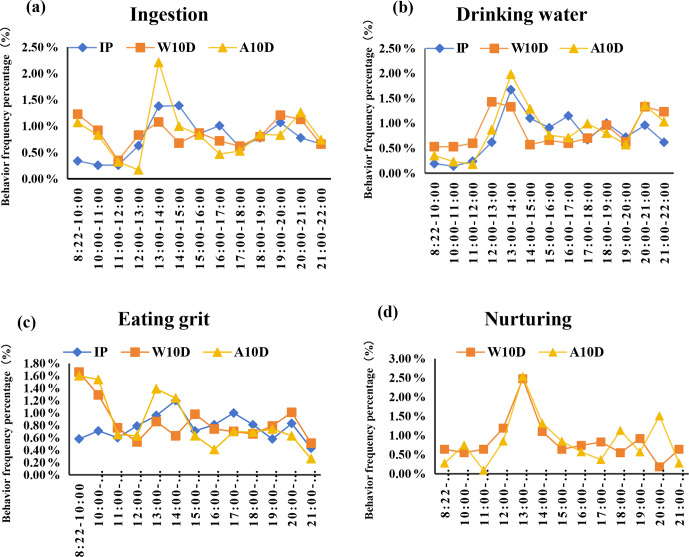
Comparison of the frequency percentage of **(a)** ingestion, **(b)** water consumption, **(c)** grit consumption, and **(d)** nurturing behavior among parent pigeons at different stages of the breeding period. Abbreviations used in the figure are as follows: IP – incubation period; W10D – within the first 10 d of the nurturing period; A10D – after 10 d during the nursing period.

According to our findings, the frequency of ingestion by parent pigeons during the incubation period fluctuated, peaking at 9.10 times per hour during the 13:00–15:00 LT period. It was also noteworthy that the frequency of ingestion was lower during the 08:22–12:00 LT period (Fig. 2a, Table S1 in the Supplement). Similarly, the water consumption frequency ( P<0.05 ; Fig. 2b, Table S1) during the 13:00–14:00 LT period was significantly higher than that during the other periods, with the next-highest period being 18:00–19:00 LT ( P<0.05 ). According to Fig. 2c and Table S1, the parents' maximum frequency of grit consumption was 5.60 times per hour between 14:00 and 15:00 LT; however, this difference did not change significantly throughout the study.

Fig. 2a and Table S1 also show that the eating frequency of parent pigeons with young that were older than 10 d changed throughout the nursing period, although the range of change was small. Of these, the highest ingestion frequency occurred between 08:22 and 10:00 LT, followed by 13:00–14:00 LT, and then 19:00–21:00 LT ( P<0.05 ). The pigeons achieved their maximum ingestion frequency between 08:22 and 10:00 LT. Additionally, there was greater uniformity in the frequency of water consumption between 12:00 and 13:00 LT ( P<0.05 ; Fig. 2b, Table S1), with the frequency of this behavior being higher during this time compared with other periods. The time period from 08:22 to 10:00 LT had the highest frequency of grit consumption, with a value of 13.11 times per hour; this was followed by 10:00–11:00, 20:00–21:00, and 15:00–16:00 LT, although there was no statistically significant difference between these time periods (Fig. 2c, Table S1). The frequency of grit consumption dropped gradually during the 15:00–19:00 LT period. As shown in Fig. 2c and Table S1, the nursing frequency was substantially higher between 13:00 and 14:00 LT, peaking at 3.00 ± 0.62 times per hour.

During the nursing period, the ingestion frequency of parent pigeons with young that were older than 10 d decreased gradually, as shown in Fig. 2a and Table S1, reaching a minimum frequency of 1.56 ± 0.50 times per hour from 08:22 to 10:00 LT. It then peaked at 13:00–14:00 LT ( P<0.05 ), which showed a significantly higher frequency of this behavior compared with other periods, and then continued to decrease. The frequency of drinking water intake increased from 20:00 to 21:00 LT, reaching 11.78 ± 3.54 times per hour, which was remarkable. Compared to the other time periods, the parents' drinking frequency was substantially higher between 13:00 and 14:00 LT ( P<0.05 ; Fig. 2b, Table S1) and recovered between 20:00 and 21:00 LT. The least amount of grit consumption happened between 16:00 and 17:00 LT ( 3.67 ± 0.97 , P<0.05 ). After that, it steadily went up and was much more common between 08:22 and 11:00 LT ( 14.4 ± 2.56 ; 13.89 ± 3.20 ) and between 13:00 and 14:00 LT ( 12.56 ± 2.48 ) compared with the other periods (Fig. 2c, Table S1). In contrast to parents who were in the first 10 d of the nurturing period, parents with young that were older than 10 d during the nursing period experienced more fluctuations in nursing frequency, peaking at 13:00–14:00 LT ( 3.00 ± 0.53 , P<0.05 ; Fig. 2d, Table S1). During the nursing period, the nursing frequency decreased gradually to a minimum of 1.56 times per hour from 08:22 to 10:00 LT.

### 3.4 The frequency of stretching, preening, and resting behaviors among pigeons during different stages of the breeding period

Table 5 displays the frequency of parent pigeons' stretching, preening, and resting activities during various breeding periods. The frequency of resting behavior for parent pigeons with young that were older than 10 d of age during the nursing period ( 63.00 ± 7.65 , P<0.05 ) was substantially higher than that of parent pigeons within the first 10 d of the nurturing period ( 30.44 ± 2.89 , P<0.05 ) and throughout the incubation period ( 20.50 ± 4.97 , P<0.05 ). However, there were no significant variations in the frequency of the parents' stretching and preening behavior at various times during the breeding period.

**Table 5:** Comparison of the total frequency of stretching, preening, and resting behaviors of parent pigeons at different times during the breeding period.

Total frequency	IP	W10D	A10D	P value
Stretching	134.40 ± 12.37	146.11 ± 15.90	135.67 ± 13.67	0.821
Preening	326.80 ± 41.41	349.89 ± 60.59	224.56 ± 20.86	0.127
Resting	20.50 ± $4.97^{b}$	30.44 ± $2.89^{b}$	63.0 ± $7.65^{a}$	0.001

^a,b^ Means within the same column differ substantially ( P<0.05 ). Abbreviations used in the table are as follows: IP – incubation period; W10D – within the first 10 d of the nurturing period; A10D – after 10 d during the nursing period. Values in the IP, W10D, and A10D columns are given in grams per day per pair.

**Figure 3:**
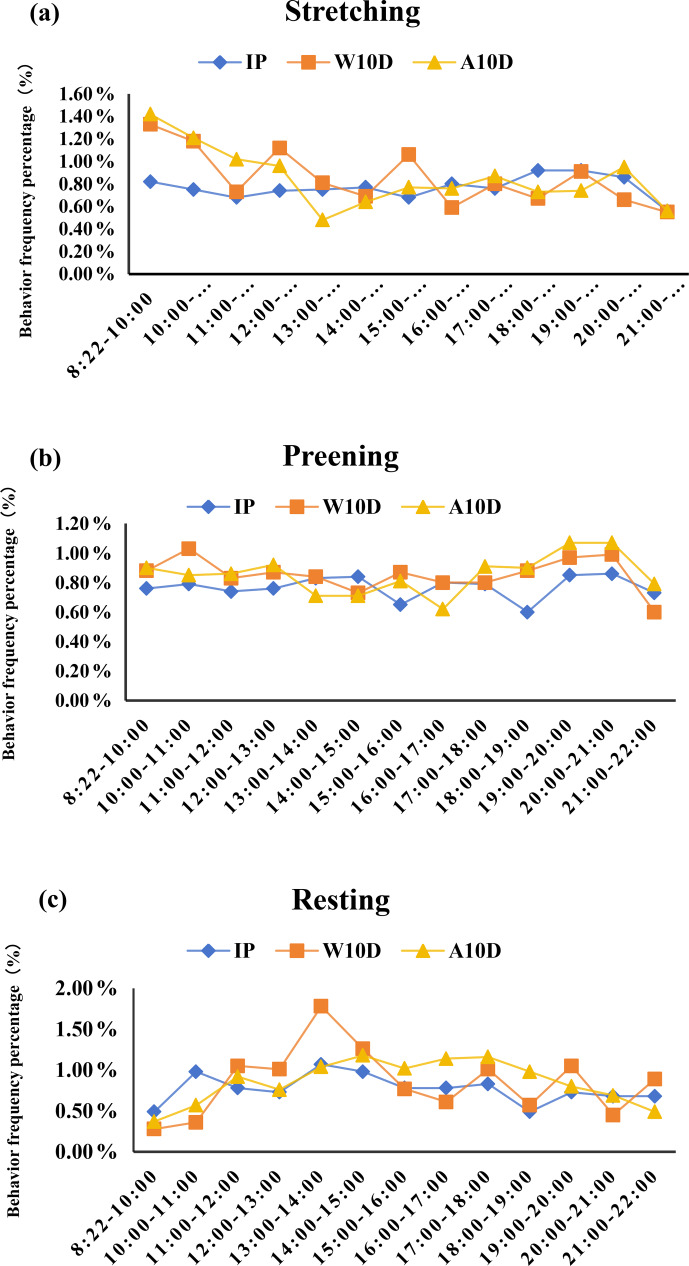
Comparison of the percentage of **(a)** stretching, **(b)** preening, and **(c)** resting behaviors of parent pigeons at different stages of the breeding period. Abbreviations used in the figure are as follows: IP – incubation period; W10D – within the first 10 d of the nurturing period; A10D – after 10 d during the nursing period.

It was less likely that frequency of stretching ( P=0.875 ; Fig. 3a, Table S2 in the Supplement), preening ( P=0.980 ; Fig. 3b, Table S2), and resting ( P=0.974 ; Fig. 3c, Table S2) behaviors changed during the incubation period. There was also no significant difference between the three behaviors during the periods. At 19:00–20:00 LT, stretching behavior peaked at 12.40 ± 1.69 times per hour, while feather grooming behavior peaked at 20:00–21:00 LT with a frequency of 28.10 ± 4.90 times per hour (Fig. 3a, Table S2). It is noteworthy to add that the maximal resting frequency value (13:00–14:00 LT; 2.20 ± 0.79 , P<0.05 ; Fig. 3c, Table S2) matched the male and female pigeons' alternate egg incubation times (12:00–13:00, 14:00–16:00, and 19:00–22:00 LT; Fig. 1).

The stretching frequency of parent pigeons throughout the first 10 d of the nursing period varied, peaking at 17.44 ± 1.95 times per hour between 08:22 and 10:00 LT. From 08:22 to 12:00 LT, the frequency of stretching decreased gradually (Fig. 3a, Table S2). With smaller fluctuations in changes and no discernible difference in fluctuations in changes, the preening frequency was higher throughout all periods ( P=0.957 ; Fig. 3b, Table S2). Resting behavior peaked between 13:00 and 14:00 LT ( P<0.05 ; Fig. 3c, Table S2); compared to all other periods, this was the only time when the resting frequency was considerably greater. From 13:00 to 17:00 LT, the resting frequency steadily dropped.

Throughout the nursing period, the frequency of stretching for parent pigeons with young that were older than 10 d varied somewhat in each period before gradually declining to the lowest frequency between 08:22 and 14:00 LT ( P=0.001 ; Fig. 3a, Table S2). During all periods, the preening frequency increased and changed less dramatically ( P=0.429 ; Fig. 3b, Table S2). The frequency of resting behavior changed somewhat between times, peaking between 14:00 and 15:00 LT ( P<0.05 ; Fig. 3c, Table S2).

### 3.5 The frequency of courtship, fighting, and mating behaviors among pigeons during different stages of the breeding period

Table 6 displays the prevalence of courtship, fighting, and mating behaviors among pigeons over various phases of the breeding cycle. The frequency of parental fighting behavior during the first 10 d of the nurturing period ( P≤0.05 ) was much higher than the frequency of fighting behavior during the later nursing period ( P≤0.05 ) or the incubation phase ( P≤0.05 ). There was a lot more courtship behavior in parents with young that were older than 10 d compared with the incubation period and the earlier nursing phase ( P≤0.05 ). There was also a lot less courtship behavior in the first 10 d of the nurturing period ( P≤0.05 ). During the incubation phase, there was no mating behavior, and the difference between the two groups – 1.33 times during the first 10 d of life and 2.00 times after the young were more than 10 d of age – was not statistically significant.

**Table 6:** Comparison of the total frequency of courtship, fighting, and mating behaviors among pigeons during different phases of the breeding period.

Total frequency	IP	W10D	A10D	P value
Fighting	26.90 ± $5.57^{b}$	62.67 ± $16.53^{a}$	31.22 ± $6.43^{b}$	0.047
Courtship	25.60 ± $6.57^{b}$	64.89 ± $13.86^{b}$	97.33 ± $25.83^{a}$	0.019
Mating	–	1.33 ± 0.50	2.00 ± 0.60	0.406

^a,b^ Means within the same column differ substantially ( P<0.05 ). Abbreviations used in the table are as follows: IP – incubation period; W10D – within the first 10 d of the nurturing period; A10D – after 10 d during the nursing period. Values in the IP, W10D, and A10D columns are given in grams per day per pair.

**Figure 4:**
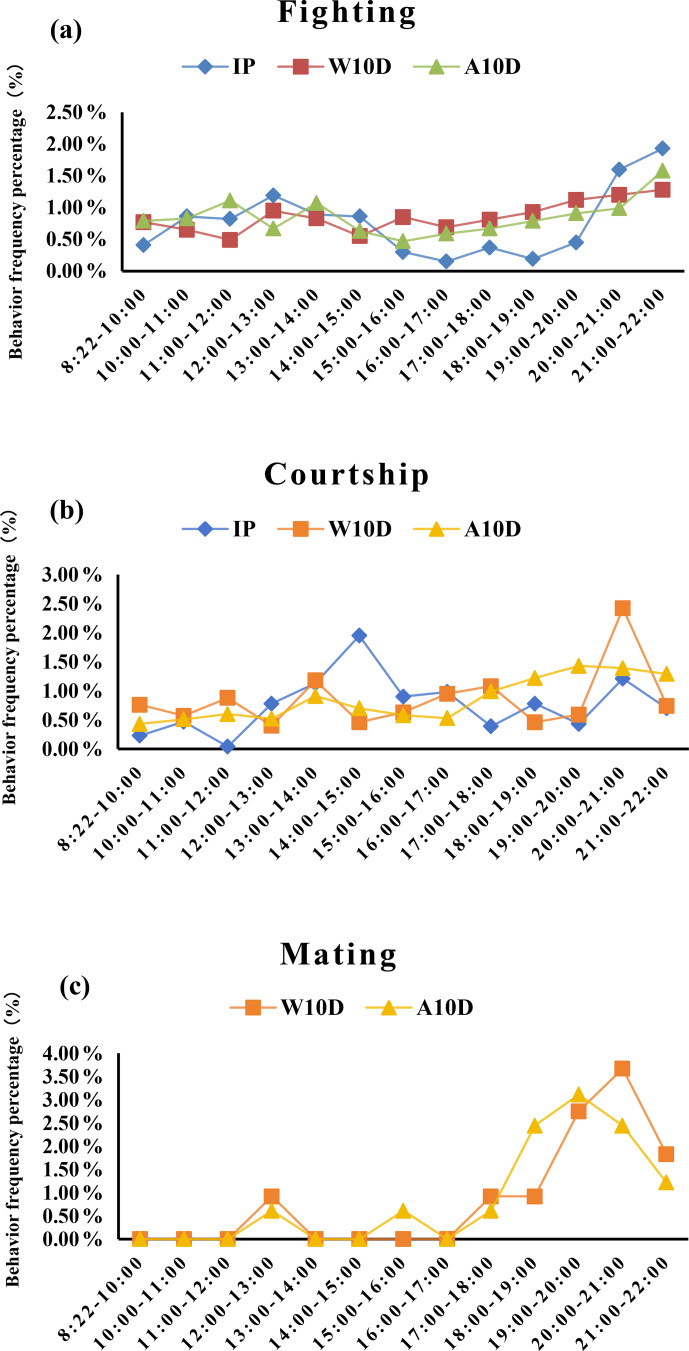
Comparison of the frequency percentage of **(a)** fighting, **(b)** courtship, and **(c)** mating behavior among pigeons at different stages of the breeding period. Abbreviations used in the figure are as follows: IP – incubation period; W10D – within the first 10 d of the nurturing period; A10D – after 10 d during the nursing period.

Fighting was much more common between 20:00 and 22:00 LT ( P<0.05 ; Fig. 4a, Table S3 in the Supplement) compared with other times during the incubation period. The frequency of courtship behavior peaked between 14:00 and 15:00 LT but did not change significantly otherwise during the incubation period ( P=0.468 ; Fig. 4b, Table S3). Figure 4c shows that the parents did not engage in any mating behavior during the incubation period.

During the first 10 d of the nursing phase, there was no significant difference in the frequency of parental fights between different time periods ( P=0.804 ; Fig. 4a, Table S3). Compared to all other time periods, the frequency of courtship behavior was substantially higher between 20:00 and 21:00 LT ( P<0.05 ; Fig. 4b, Table S3). However, parental mating behavior primarily occurred after 17:00 LT and reached a maximum frequency of 0.44 times per hour during the time period from 20:00 to 21:00 LT (Fig. 4c, Table S3). It is important to note that fighting and courtship also occur during mating behavior.

After young were 10 d of age during the nursing phase, variations in the frequency of parent pigeons' fighting and courtship behavior among time periods were insignificant ( P=0.557 , P=0.197 ; S3). However, the frequency of conflicts and courtship moments increased after 17:00 LT. Between 19:00 and 20:00 LT, the mating behavior of parent pigeons reached an apex ( P<0.05 ; Fig. 4c, Table S3) and was noticeably higher compared with other time periods. On the other hand, compared to all other times when mating behavior took place, courtship was more common after 17:00 LT (Fig. 4c).

## 4 Discussion

Our results show the incubation phase of brood pigeons, with males dominating from 13:00 to 20:00 LT and females continuing after 20:00 LT. The average incubation period was 7.94 h for females and 4.05 h for males, with females taking 96.05 % longer to incubate.

Pigeons incubate their eggs by nestling them in the exposed space under their abdomen, akin to sleeping. Regardless of whether the female or male is brooding, parent pigeons roll the eggs under their beaks and feet during the incubation phase to make sure the eggs are fully covered (Deeming and Reynolds, 2015). Around this time, females primarily begin incubating eggs (Archer and Cartwright, 2012; Abou-Kassem et al., 2024). The jambu fruit dove's mating habits are characterized by a diurnal rhythm: the female incubates for longer periods than the male, especially during the night and throughout the entire morning, after the male finishes his incubation period at noon (Kozlowski et al., 2016; Wan et al., 2018).

Our findings align with prior research, indicating that females incubate eggs for a longer duration than males. To avoid unequal heat exposure that could lead to hatching failure, breeders should offer comfortable nests, support the parents' incubation postures, and help retain heat. Feathers and cotton wool can be added to the cage to encourage the parent pigeons' natural behaviors, such as roosting and nest building.

For parent pigeons with offspring more than 10 d old during the nurturing period, our results show that the frequency of feeding between 13:00 and 14:00 LT significantly increases and that nurturing behavior concurrently reaches its daily peak. During the nursing period, pigeons consume more grit and water. Ingestion frequency decreases during the nursing period, whereas FI and drinking frequency increase. Grit consumption also steadily increases. During the nursing period, the overall ingestion frequency and FI are both higher than those during the incubation period. The parent pigeons' nursing frequency correlates with the growth of squabs, as they require more frequent feeding to sustain the young (Kozlowski et al., 2016). According to research by Zhang et al. (2023), Tarim pigeons' absolute growth with respect to body weight increases steadily from 0 to 10 d of age, peaking at 10 d. When fed crop milk, fledglings grow rapidly (Jin et al., 2023; Wang et al., 2023), demonstrating that this quick growth stage is crucial for them to attain a normal adult pigeon size. At first, after hatching, the fledglings' diet consists entirely of crop milk, but as they mature, they steadily increase the number of grains in their diet while decreasing the amount of crop milk (Murton et al., 1964; Stocks, 2021). Compared to the early nursing period and the incubation period, the amount of feed that parents give their fledglings during the nursing period increases significantly once the young are 10 d old due to their growing nutritional needs. Moreover, the higher percentage of grains in the diet of juvenile pigeons can reflect their improved capacity to consume greater amounts of feed as they age (Peng et al., 2023; Adawy and Abdel-Wareth, 2023).

In general, morning and evening are when birds feed the most. Allen (2009) states that, in the wild, birds not involved in incubation behavior depart to feed after an incubation exchange. Incubating birds may feed irregularly at the start of the incubation period. After that, they mainly feed sporadically throughout the day, with feeding peaks characterized by an increased duration and frequency of feeding (Tulp and Schekkerman, 2006; Boucaud et al., 2016). During the nurturing phase, parent pigeons exhibit a higher feeding frequency at 19:00 LT, a feeding behavior that aligns with their circadian rhythm. When temperatures and sunshine levels drop quickly, pigeon-feeding activity peaks. According to Aulsebrook et al. (2021) and Nissa et al. (2024), all birds exhibit two peaks in their feeding activity, with the maximum levels happening in the morning and evening. Birds are active during the light phase and quiescent during the dark phase. Parent pigeons must secrete crop milk during the nursing stage to feed their squabs, necessitating a greater protein and energy intake. As a result, after 19:00 LT, the frequency of feeding remains high. Parent pigeons eat more frequently between 08:22 and 10:00 LT. They feed their young squabs more frequently in the early stages of upbringing, from 08:22 to 10:00 LT in the morning, than when the squabs are older than 10 d. The squabs' younger age, which necessitates more frequent feeding by the parent pigeons, could be the cause of this phenomenon (Allen, 2009). It also takes longer for more frequent feedings, as parent pigeons must incubate their young and provide warmth and cover (Blechman, 2007). It is noteworthy that after artificial feeding of raw grains, the frequency of ingestion behavior significantly increases across three different stages of the breeding period. The nurturing behavior peaks between 12:00 and 14:00 LT, indicating that artificial feeding influences chick-rearing behavior.

Ingested grit may provide minerals like calcium, which grains practically lack (Downs et al., 2019). Choosing calcareous grit is especially important when the birds are laying eggs (Wooten and Werner, 2004). Studies have revealed a correlation between birds' grit consumption and their diet, especially when they consume grains and other tough feeds or experience fluctuations in their mineral needs. Due to their lack of teeth or grinding surfaces, birds must rely on grit to grind or break down feed to improve nutrient absorption (Downs et al., 2019).

As pigeons lack teeth, they grind their food using gizzard grit, just like chickens do (Takasaki and Kobayashi, 2020). Additionally, the mechanical grinding of grit can eliminate parasites stuck to the stomach wall (Robinson et al., 2008). Pigeons use grit in tiny amounts, but it is an essential feed and growth enhancer during their development. Compared to the first 10 d of the nurturing and incubation periods, parent pigeons with young older than 10 d during the nursing period have the highest overall frequency of grit consumption. This is consistent with the higher FI during those periods, suggesting a relationship between FI and grit demand. According to Maya-García et al. (2021), hummingbird juveniles use a higher percentage of grit, suggesting that grit is important for grinding feed during this rapid growth and development stage. The peak times for ingestion, grit consumption, and nurturing behavior for pigeons with young older than 10 d during the nursing phase are from 13:00 to 14:00 LT. Pigeon milk gradually changes to a sole reliance on grain feed as the chicks grow through the “mid-chick” period (Murton et al., 1964), suggesting that older chicks – those older than 10 d – need more healthy grit for grinding feed.

Pigeons eat and drink more often during the incubation phase, with peak ingestion and drinking behavior observed. The 08:22–11:00 LT nursing session also demonstrates this, indicating a consistent pattern of nutritional replenishment. Before the peak feeding times, the breeder should provide feed, grit, and water in the morning and evening. During these peak feeding times, the breeder should minimize entering the loft to avoid frightening the parent pigeons, which could disrupt their original behavioral patterns and impact their normal growth and development.

Our findings show that pigeons engage in more stretching, preening, and resting during the mating season than during incubation and nursing periods. However, parental behavior remains the same. During nursing, stretching frequencies peak, whereas resting and preening frequencies remain constant. Once the young are older than 10 d, the frequency of stretching behavior in parent pigeons decreases.

The existence, persistence, and distribution of feathers on the body are markers of birds' health, behavior, and nutritional status (Heerkens et al., 2014; Nicol, 2019). Birds frequently nibble their feathers gently (Kozlowski, 2016). Preening behavior is primarily associated with dust-bathing and remains relatively consistent over time (Baxter et al., 2018). A bird can alter the position of its feathers and secrete oils during preening (Moreno-Rueda, 2017; Alves Soares et al., 2024), which also helps to clean the feathers of debris or parasites (Terrill and Shultz, 2023) and makes them waterproof (Lovette and Fitzpatrick, 2016). Each bird can shake its body and then use its beak to preen the feathers on its exterior successively. When pigeons preen, the feather shaft can bend and hold elastic potential energy because of the support provided by their cam-shaped beak (Zhao et al., 2020). The frequency of parent pigeons' preening behavior does not significantly vary throughout the breeding season. In the Northern Hemisphere, July to December is the main molting season for captive rock pigeons (Mallet-Rodrigues, 2012). This experiment was mainly conducted in August, possibly coinciding with the pigeons' molting period, which could explain the relatively high frequency of parental preening observed throughout the day during the three periods. The highest frequency of parental preening occurred during the nursing period when young were under 10 d of age, specifically between 19:00 and 21:00 LT. This was also the peak time for courting and mating behavior. Courtship activities may increase the frequency of preening, resulting in scattered feathers.

According to Gregory (2008), stretching usually occurs after resting, at the start of a new activity, and even during dust baths. Observers noticed a minor variation in the total frequency of stretching and grooming behaviors among breeding parents over time, although there was no significant difference. Likely, the parents' inability to fly due to their cage confinement caused them to stretch by fluttering their wings and doing other things. During the incubation period, there was no discernible variation in the stretching behavior. Stretching started between 08:22 and 10:00 LT during the crop-milk-feeding period when the pigeons were 2 d old, which signaled the beginning of their daily behavioral pattern. Miller et al. (2020) observed resting parents standing or reclining outside the incubation zone. During the mating season, breeding activities may alter a pigeon's resting habits. Resting more often was usual in warmer months, such as August.

Compared to the other two breeding phases, there was a considerable increase in resting frequency of parent pigeons with young that were over 10 d of age during the nursing period. The rotation of males and females during incubation may have contributed to the peak resting behavior, which did not significantly vary throughout the incubation period and peaked between 13:00 and 14:00 LT. Grooming and the length of rest had a direct correlation (Dijkstra et al., 2010; Wang et al., 2023). Feeding came after resting and grooming more frequently than during other times. During the feeding phase, resting behavior rose following the feeding peak.

According to our research, parent pigeons exhibit more fighting behavior during the first 10 d of their offsprings' life than during the nursing and incubation phases. After the young reach 10 d of age, parental courting behavior increased, and fighting was more common between 20:00 and 22:00 LT. The peak period with respect to mating was from 19:00 to 20:00 LT.

Male pigeons fight with other males in nearby cages due to their defensive behavior, protective behavior with respect to their chicks, or territorial defense. Conflicts typically occur in the area where pigeons nest, and they will defend their territory if threatened (Matos and McGregor, 2002; Grodwohl, 2019). The birds peck at feathers (Brunberg et al., 2011), go across to neighboring cages (Tablante et al., 2000), act aggressively (Campderrich et al., 2017), and peck at toes (Krause et al., 2011; Elsherbeni et al., 2024). When they get close, parent pigeons will raise their feathers, signaling that they want to attack, and may use their wings or beaks to strike or peck to defend eggs or squabs (Sax, 2017). Compared to the other two breeding stages, the overall frequency of parental courting behaviors during the first 10 d of the nurturing period is much higher. Birds peck at the tips of other birds' tail feathers for extended periods (Rodenburg et al., 2013). Like parents feeding their offspring, the male opens its beak, and the female puts hers inside the male's beak, twisting and swaying their necks to exchange food (Adawy et al., 2023). Courtship rarely happens once incubation has started in pigeons, and the parents may start a new reproductive cycle involving courtship, nesting, and mating before the young leave the nest (Goerlich-Jansson et al., 2013). The experiment's findings indicate that the total frequency of parental courtship during the nursing period was significantly higher when squabs were older than 10 d than when squabs were younger than 10 d during both the nursing and incubation periods, with the incubation period recording the lowest total frequency of courtship. Fighting behavior peaked during the incubation period between 21:00 and 22:00 LT. However, the frequency of courting behavior did not significantly vary throughout the day. Parent pigeons with young in their first 10 d of life have a much higher frequency of fighting behavior than those within the other two breeding phases. However, this difference is not statistically significant over a range of periods. Ware et al. (2017) linked the onset of mating behavior in pigeons, typically pecking with the beak and chirping, to the onset of fighting behavior. Competition for mates may have triggered fighting behavior during this period. The frequency of fighting and friendly behaviors among parent pigeons with squabs older than 10 d throughout the nursing period did not significantly differ over different time intervals during the nurturing phase. On the other hand, the peak periods of pigeon mating behavior corresponded to the times when courting behavior most frequently occurred. Mutual feeding behavior during courting typically takes place prior to mating, with preening generally occurring after mating behavior (Strycker, 2015). To avoid any detrimental effects on mating behavior and egg-laying rates, breeders should vacate the loft before the peak mating period to reduce disturbances.

## 5 Conclusion

Based on the conditions of this experiment, the subsequent conclusions may be inferred:

1. Female pigeons undertake the primary hatching task during the incubation period. Throughout the observations, female pigeons incubated the eggs for an average of 7.94 h, primarily from 08:22 to 13:00 LT and from 20:00 to 22:00 LT, whereas male pigeons' average incubation duration was 4.05 h, mainly from 13:00 to 20:00 LT.
2. During the nursing period, the feed intake and ingestion frequency values of the parent pigeons were higher compared to those during the incubation period, and these metrics increased as the squabs aged.
3. During various breeding phases, the peak times for feeding behavior occurred from 08:22 to 10:00 LT, from 13:00 to 14:00 LT, and from 19:00 to 21:00 LT. The peak times for drinking behavior were from 13:00 to 14:00 LT and from 20:00 to 21:00 LT. Nurturing behavior primarily occurred between 13:00 and 14:00 LT. In contrast, mating behavior mainly took place from 17:00 to 22:00 LT.
4. Artificial feeding affects the behavioral rhythms of pigeons, particularly the feeding and nursing behaviors.

Overall, the behaviors of parent pigeons throughout various stages of the breeding cycle can assist breeders in gaining a deeper understanding of pigeon behaviors, enhancing their management of breeding pigeons in captivity, and ultimately boosting breeding efficiency and production outcomes.

## Supplement

10.5194/aab-68-395-2025-supplement The supplement related to this article is available online at https://doi.org/10.5194/aab-68-395-2025-supplement.

## Data Availability

The datasets supporting this study have been deposited in the Mendeley Data repository (Elsevier's certified repository) with the persistent identifier 10.21203/rs.3.rs-4915351/v1 (Zhu, 2024). All data files are publicly accessible without restrictions.
