# Supplementary material for: Investigation of the reproductive behavior of Tarim pigeons
Source: Arch Anim Breed. 2025 Jun 19;68(2):395–407. doi: 10.5194/aab-68-395-2025 (PMC13283315; doi:10.5194/aab-68-395-2025)
Supplement: The supplement related to this article is available online at https://doi.org/10.5194/aab-68-395-2025-supplement. [file aab-68-395-2025-supplement.pdf]

Supplement of Arch. Anim. Breed., 68, 395–407, 2025
https://doi.org/10.5194/aab-68-395-2025-supplement
© Author(s) 2025. CC BY 4.0 License.

Open Access
Archives Animal Breeding

Supplement of

Investigation of the reproductive behavior of Tarim pigeons

Lin Zhu et al.

Correspondence to: Fengming Li (lifming@xjau.edu.cn)

The copyright of individual parts of the supplement might differ from the article licence.

Table S1. Comparison of the frequency of ingestion, drinking water, eating grit, and nurturing behavior among parent pigeons at different stages of the breeding period

**Period**	**IP**			**W10D**				**A10D**			
	**Ingestion**	**Drinking water**	**Eating grit**	**Ingestion**	**Drinking water**	**Eating grit**	**Nurturing**	**Ingestion**	**Drinking water**	**Eating grit**	**Nurturing**
8:22-10:00	2.20±0.53^{bc}	0.40±0.16^{d}	2.70±0.76	11.44±2.60^{a}	1.78±0.40^{b}	13.11±2.26^{a}	0.78±0.15^{bc}	10.00±2.18^{bc}	1.67±0.53^{def}	14.44±2.56^{a}	0.33±0.17^{cd}
10:00-11:00	1.70±0.54^{c}	0.30±0.15^{d}	3.30±1.45	8.56±2.08^{abc}	1.78±0.60^{b}	10.22±2.85^{ab}	0.67±0.29^{bc}	7.78±2.89^{bcd}	1.11±0.39^{ef}	13.89±3.20^{a}	0.89±0.39^{bcd}
11:00-12:00	1.70±0.75^{c}	0.50±0.17^{cd}	2.80±0.63	3.22±0.93^{c}	2.00±0.58^{b}	6.00±1.38^{b}	0.78±0.28^{bc}	3.00±0.93^{cd}	0.89±0.26^{f}	5.89±1.11^{bc}	0.11±0.11^{d}
12:00-13:00	4.10±1.92^{abc}	1.30±0.40^{bcd}	3.70±1.09	7.67±1.79^{abc}	4.78±1.66^{a}	4.22±1.01^{b}	1.44±0.48^{b}	1.56±0.50^{d}	4.22±1.01^{bcdef}	5.67±1.70^{bc}	1.00±0.24^{bcd}
13:00-14:00	9.00±2.22^{a}	3.50±0.89^{a}	4.50±1.29	10.00±1.89^{ab}	4.44±0.78^{ab}	6.78±1.76^{b}	3.00±0.62^{a}	20.67±3.62^{a}	9.56±1.35^{a}	12.56±2.48^{a}	3.00±0.53^{a}
14:00-15:00	9.10±2.42^{a}	2.30±0.50^{ab}	5.60±1.02	6.33±0.75^{abc}	1.89±0.51^{b}	5.00±2.30^{b}	1.33±0.41^{bc}	9.33±3.03^{bcd}	6.22±1.34^{abc}	11.22±3.46^{ab}	1.56±0.65^{bc}
15:00-16:00	5.60±1.38^{abc}	1.90±0.43^{bc}	3.30±1.14	8.11±1.38^{abc}	2.22±0.60^{ab}	7.78±3.06^{ab}	0.78±0.22^{bc}	7.78±3.04^{bcd}	3.67±0.97^{bcdef}	5.67±1.40^{bc}	1.00±0.37^{bcd}
16:00-17:00	6.60±1.75^{abc}	2.40±0.58^{ab}	3.80±1.08	6.67±1.94^{abc}	2.00±0.73^{b}	5.89±1.95^{b}	0.89±0.26^{bc}	4.44±1.87^{bcd}	3.44±0.44^{bcdef}	3.67±0.97^{c}	0.67±0.24^{bcd}
17:00-18:00	3.90±0.53^{bc}	1.40±0.27^{bcd}	4.70±1.80	5.78±1.38^{bc}	2.33±0.50^{ab}	5.56±1.17^{b}	1.00±0.44^{bc}	5.00±1.63^{bcd}	4.78±1.82^{bcde}	6.33±1.23^{bc}	0.44±0.24^{bcd}
18:00-19:00	5.10±1.38^{abc}	2.10±0.61^{ab}	3.80±0.99	7.44±1.57^{abc}	3.22±0.88^{ab}	5.22±0.94^{b}	0.67±0.24^{bc}	8.00±2.40^{bcd}	3.89±0.94^{bcdef}	6.22±1.06^{bc}	1.33±0.76^{bcd}
19:00-20:00	7.00±1.81^{ab}	1.50±0.40^{bcd}	2.70±0.79	11.22±1.65^{ab}	2.11±0.42^{ab}	6.22±1.83^{b}	1.11±0.35^{bc}	7.78±2.48^{bcd}	2.78±0.76^{cdef}	6.67±1.73^{bc}	0.67±0.37^{bcd}
20:00-21:00	5.10±1.36^{abc}	2.00±0.42^{b}	3.90±0.92	10.44±1.80^{ab}	4.44±1.30^{ab}	8.00±2.70^{ab}	0.22±0.22^{c}	11.78±3.54^{b}	6.56±1.38^{ab}	5.67±1.92^{bc}	1.78±0.52^{b}
21:00-22:00	4.30±1.63^{abc}	1.30±0.50^{bcd}	2.00±0.67	6.11±1.07^{abc}	4.11±0.86^{ab}	4.00±1.20^{b}	0.78±0.22^{bc}	6.89±2.30^{bcd}	5.00±1.99^{bcd}	2.33±0.69^{c}	0.33±0.24^{cd}
P- value	0.005	<0.001	0.671	0.027	0.033	0.099	<0.001	<0.001	<0.001	<0.001	<0.001

^{a,b,c,d,f} Means within the same column differ substantially ($p<0.05$).
IP= incubation period; W10D= within the first 10 days of the nurturing period; A10D= above 10 days of age during the nursing period.

Table S2. Comparison of the frequency of stretching, preening, and resting behaviors of parent pigeons at different stages of the breeding period

Period	IP			W10D			A10D		
	**Stretching**	**Preening**	**Resting**	**Stretching**	**Preening**	**Resting**	**Stretching**	**Preening**	**Resting**
8:22-10:00	11.00±1.19	24.70±5.22	1.00±0.49	17.44±1.95^{a}	27.67±4.97	0.78±0.32^{d}	17.33±2.85^{a}	18.11±3.39^{ab}	2.11±0.87^{d}
10:00-11:00	10.10±2.37	25.90±4.33	2.00±0.75	15.56±2.43^{ab}	32.44±7.00	1.00±0.29^{cd}	14.78±1.88^{ab}	17.22±2.09^{ab}	3.22±0.78^{bcd}
11:00-12:00	9.10±2.06	24.30±3.96	1.60±0.50	9.56±1.73^{bc}	26.11±4.11	2.89±0.48^{bc}	12.44±1.87^{abc}	17.44±2.67^{ab}	5.22±1.35^{abcd}
12:00-13:00	9.90±1.77	24.80±2.04	1.50±0.48	14.78±3.32^{abc}	27.44±4.10	2.78±0.88^{bc}	11.67±1.78^{bcd}	18.56±3.41^{ab}	4.33±1.50^{abcd}
13:00-14:00	10.10±2.18	27.10±4.41	2.20±0.79	10.67±1.05^{abc}	26.44±4.71	4.89±1.09^{a}	5.89±0.81^{d}	14.33±2.72^{ab}	5.89±0.84^{abc}
14:00-15:00	10.40±2.27	27.40±4.41	2.00±0.89	9.11±3.04^{bc}	23.11±4.95	3.44±0.53^{ab}	7.78±1.62^{cd}	14.44±2.55^{ab}	6.67±1.17^{a}
15:00-16:00	9.10±1.51	21.30±3.75	1.60±0.48	14.00±2.52^{abc}	27.33±5.00	2.11±0.48^{bcd}	9.44±1.94^{bcd}	16.44±2.53^{ab}	5.78±0.97^{abc}
16:00-17:00	10.70±1.68	26.20±4.74	1.60±0.52	7.78±1.49^{c}	25.22±5.90	1.67±0.53^{bcd}	9.33±2.12^{bcd}	12.44±1.80^{b}	6.44±0.88^{ab}
17:00-18:00	10.20±1.53	25.90±5.66	1.70±0.52	10.56±2.12^{abc}	25.33±4.40	2.78±0.57^{bc}	10.67±1.34^{bcd}	18.33±1.83^{ab}	6.56±1.17^{a}
18:00-19:00	12.30±1.56	19.50±2.51	1.00±0.26	8.78±2.54^{bc}	27.78±5.43	1.56±0.29^{bcd}	8.89±1.18^{cd}	18.11±2.62^{ab}	5.56±0.85^{abc}
19:00-20:00	12.40±1.69	27.80±5.08	1.50±0.58	12.00±2.19^{abc}	30.67±7.62	2.89±0.63^{bc}	9.00±1.38^{bcd}	21.67±1.97^{a}	4.56±0.78^{abcd}
20:00-21:00	11.60±1.83	28.10±4.90	1.40±0.48	8.67±2.13^{bc}	31.33±8.03	1.22±0.32^{cd}	11.56±2.26^{bcd}	21.56±3.07^{a}	3.89±0.81^{abcd}
21:00-22:00	7.50±1.44	23.80±3.79	1.40±0.65	7.22±2.07^{c}	19.00±3.25	2.44±0.53^{bcd}	6.89±1.23^{cd}	15.89±2.70^{ab}	2.78±0.68^{cd}
P-value	0.875	0.980	0.974	0.027	0.957	＜0.001	0.001	0.429	0.014

^{a,b,c,d} Means within the same column differ substantially (P<0.05).

IP= incubation period; W10D= within the first 10 days of the nurturing period; A10D= above 10 days of age during the nursing period.

Table S3. Comparison of the frequency of fighting, intimacy, and mating behavior among pigeons at different stages of the breeding period

Period	IP		W10D			A10D		
	Fighting	Intimacy	Fighting	Intimacy	Mating	Fighting	Intimacy	Mating
8:22-10:00	1.10±0.28[cd]	0.60±0.31[b]	4.33±1.18	4.44±1.86[b]	0.00±0.00[b]	2.22±0.80[ab]	3.78±2.04	0.00±0.00[b]
10:00-11:00	2.30±0.60[bcd]	1.20±0.73[ab]	3.67±1.00	3.33±1.60[b]	0.00±0.00[b]	2.33±0.58[ab]	4.44±2.62	0.00±0.00[b]
11:00-12:00	2.20±0.57[bcd]	0.10±0.10[b]	2.78±1.04	5.11±2.40[b]	0.00±0.00[b]	3.11±0.75[ab]	5.22±3.11	0.00±0.00[b]
12:00-13:00	3.20±1.07[abc]	2.00±1.37[ab]	5.33±1.51	2.33±0.97[b]	0.11±0.11[ab]	1.89±0.51[ab]	4.56±1.87	0.11±0.11[b]
13:00-14:00	2.40±0.81[bcd]	2.90±1.72[ab]	4.67±1.60	6.89±2.07[b]	0.00±0.00[b]	3.00±1.03[ab]	8.00±2.19	0.00±0.00[b]
14:00-15:00	2.30±1.09[bcd]	5.00±2.15[a]	3.11±1.25	2.67±2.19[b]	0.00±0.00[b]	1.78±0.55[ab]	6.11±3.16	0.00±0.00[b]
15:00-16:00	0.80±0.36[cd]	2.30±1.56[ab]	4.78±2.25	3.67±1.75[b]	0.00±0.00[b]	1.33±0.47[b]	5.11±1.97	0.11±0.11[b]
16:00-17:00	0.40±0.31[d]	2.50±1.97[ab]	3.89±1.41	5.56±1.90[b]	0.00±0.00[b]	1.67±0.44[ab]	4.67±2.89	0.00±0.00[b]
17:00-18:00	1.00±0.47[cd]	1.00±0.70[ab]	4.56±1.69	6.33±2.16[b]	0.11±0.11[ab]	1.89±0.70[ab]	8.67±2.57	0.11±0.11[b]
18:00-19:00	0.50±0.34[d]	2.00±1.37[ab]	5.22±2.56	2.67±0.96[b]	0.11±0.11[ab]	2.22±0.66[ab]	10.67±1.61	0.44±0.24[ab]
19:00-20:00	1.20±0.55[cd]	1.10±0.53[ab]	6.33±2.21	3.44±1.43[b]	0.33±0.33[ab]	2.56±0.85[ab]	12.56±3.13	0.56±0.24[a]
20:00-21:00	4.30±1.34[ab]	3.10±1.07[ab]	6.78±2.16	14.11±4.42[a]	0.44±0.18[a]	2.78±1.06[ab]	12.22±3.99	0.44±0.24[ab]
21:00-22:00	5.20±1.05[a]	1.80±1.05[ab]	7.22±1.13	4.33±1.78[b]	0.22±0.15[ab]	4.44±1.79[a]	11.33±3.84	0.22±0.15[ab]
P- value	<0.001	0.468	0.804	0.022	0.180	0.557	0.195	0.015

[a,b,c,d] Means within the same column differ substantially ($P<0.05$).

IP= incubation period; W10D= within the first 10 days of the nurturing period; A10D= above 10 days of age during the nursing period.
